# Understanding of MoS_2_/GaN Heterojunction Diode and its Photodetection Properties

**DOI:** 10.1038/s41598-018-30237-8

**Published:** 2018-08-07

**Authors:** Monika Moun, Mukesh Kumar, Manjari Garg, Ravi Pathak, Rajendra Singh

**Affiliations:** Department of Physics, Indian Institute of Technology Delhi, New Delhi, 110016 India

## Abstract

Fabrication of heterojunction between 2D molybdenum disulfide (MoS_2_) and gallium nitride (GaN) and its photodetection properties have been reported in the present work. Surface potential mapping at the MoS_2_/GaN heterojunction is done using Kelvin Probe Force Microscopy to measure the conduction band offset. Current-voltage measurements show a diode like behavior of the heterojunction. The origin of diode like behavior is attributed to unique type II band alignment of the heterojunction. The photocurrent, photoresponsivity and detectivity of the heterojunction are found to be dependent on power density of the light. Photoresponse investigations reveal that the heterojunction is highly sensitive to 405 nm laser with very high responsivity up to 10^5^ A/W. The heterojunction also shows very high detectivity of the order of 10^14^ Jones. Moreover, the device shows photoresponse in UV region also. These observations suggest that MoS_2_/GaN heterojunction can have great potential for photodetection applications.

## Introduction

Two dimensional transition metal dichalcogenides (TMDCs) have garnered a great research interest due to their unique electrical, mechanical, optical and chemical properties making them preferable for potential applications in electronic and optoelectronic devices^1–3^. Weak interlayer van der Waals interaction present in TMDCs facilitates the exfoliation of bulk crystal in few layers offering layer dependent unique properties^4,5^. Since most of the 2D materials have relatively smaller bandgap, optoelectronic and photovoltaic devices based on these semiconductors can be realized due to their excellent light absorption properties^6–8^. Molybdenum disulfide (MoS_2_) is a typical layered transition metal dichalcogenide having indirect bandgap of 1.2 eV in bulk form and direct bandgap of 1.8 eV when it is in monolayer form^4,9^. Due to its unique layer dependent appealing properties such as high mobility and excellent light absorption covering broad range of spectral response, it has been extensively studied by scientists for various applications such as field effect transistors^10,11^, gas sensors^12,13^, photodetectors^14–16^ and flexible devices^17^. Moreover, researchers have shown their keen interest in observing photoresponse in MoS_2_ based photovoltaic and optoelectronic devices^18–20^. The first optoelectronic device based on monolayer MoS_2_ was a phototransistor with photoresponsivity of 7.5 mA/W fabricated by Yin *et al*.^19^. High photoresponsivity of 880 A/W much higher than first graphene photodetector^21^ (0.5 mA/W) was achieved on mechanically exfoliated monolayer MoS_2_ based photodetector^14^. Multilayer MoS_2_ has also been considered in optically active devices for example, Kim *et al*. explored the optoelectronic properties of TFTs based on multilayer MoS_2_ and showed that it can be used in high detectivity phototransistors^22^.

Absence of dangling bonds in 2D layered materials facilitates their integration with three dimensional semiconductors to form van der Waals heterostructures. To further explore the properties of 2D layered materials for applications in nanoscale electronic and photovoltaic devices, their integration with bulk semiconductors has been explored in recent years utilizing the advantages of both 2D and 3D materials^23–31^. Heterogeneous integration of MoS_2_ with bulk materials such as Si, GaN^32^, SiC^33^ and SnO^34^ has been studied in recent past demonstrating the promising application of 2D/3D heterostructure based devices.

Considering MoS_2_/GaN heterojunction, GaN being a wide bandgap semiconductor faces challenges in p-type doping. Integration of narrow bandgap semiconductors with GaN can lead to high performance devices but their performance gets limited due to lattice mismatch issue. This constraint can be solved by use of TMDCs such as MoS_2_. In addition to it, heterojunction of MoS_2_ and GaN can be a potential candidate in heterojunction bipolar transistor device. One of the important consequence of MoS_2_/GaN heterojunction is the enhancement in the photoresponse. Wide bandgap materials such as GaN are used in UV photodetection whereas MoS_2_ can show response varying from visible to near infrared region since its bandgap lies in the range 1–2 eV. Thus, broadband photodetection can be achieved by integrating GaN with MoS_2_.

In the recent years, a few reports have investigated MoS_2_/GaN heterojunction as a promising platform for electronic devices^35–37^. Duan *et al*. reported strong enhancement of electroluminescence in vertically stacked MoS_2_/GaN heterostructure which is difficult to achieve in other traditional indirect bandgap semiconductors^36^. Semiconductor-insulator-semiconductor diode consisting of MoS_2_, h-BN and GaN has been demonstrated by Jeong *et al*. exhibiting diode like characteristics and photoresponsivity of 1.2 mA/W^37^. These investigations demonstrated that MoS_2_/GaN heterojunctions have great potential in high performance electronic devices.

In the present work, fabrication of exfoliated MoS_2_/GaN heterojunction and its characterization using KPFM and current-voltage (I-V) measurements have been reported. Determination of parameters such as work function difference and conduction band offset play important role to understand the charge transport in 2D/3D heterojunction. We focus on understanding of type II band alignment at the interface of MoS_2_/GaN heterojunction. Further, we emphasize on understanding photoresponse behavior of the heterojunction and show that heterojunction of multilayer MoS_2_ with GaN can be used in optoelectronic applications. Detailed investigation of band offset and high photoresponsivity of MoS_2_/GaN heterojunction can open a pathway for the integration of dissimilar semiconductors. This may lead to high performance, energy efficient optoelectronic devices, also their incorporation can enhance the functionality of both wide band gap semiconductors and 2D layered semiconductors.

## Results

The most common method for growth of MoS_2_ in the fabrication of 2D/3D heterostructures is the chemical method whereas mechanically exfoliated MoS_2_ is reported to have excellent crystalline nature with less defects and can offer excellent device properties as compared to chemically synthesized MoS_2_. The presence of MoS_2_ flakes exfoliated from bulk crystal onto GaN substrate was confirmed using Raman spectroscopy and thickness was measured using AFM (shown in supporting Fig. S1). The dominant modes of vibrations which are usually observed in Raman spectra are E_2g_^1^ and A_1g_ indicating in plane and out of plane vibrations, respectively. The E_2g_^1^ and A_1g_ modes are located at 383.8 cm^−1^ and 408.3 cm^−1^, respectively. The difference between the Raman peaks is measured to be 24.5 cm^−1^ which indicates the existence of multilayer MoS_2_^38^.

### Electrical characterization of exfoliated MoS_2_/n-GaN heterojunction

**Figure 1:**
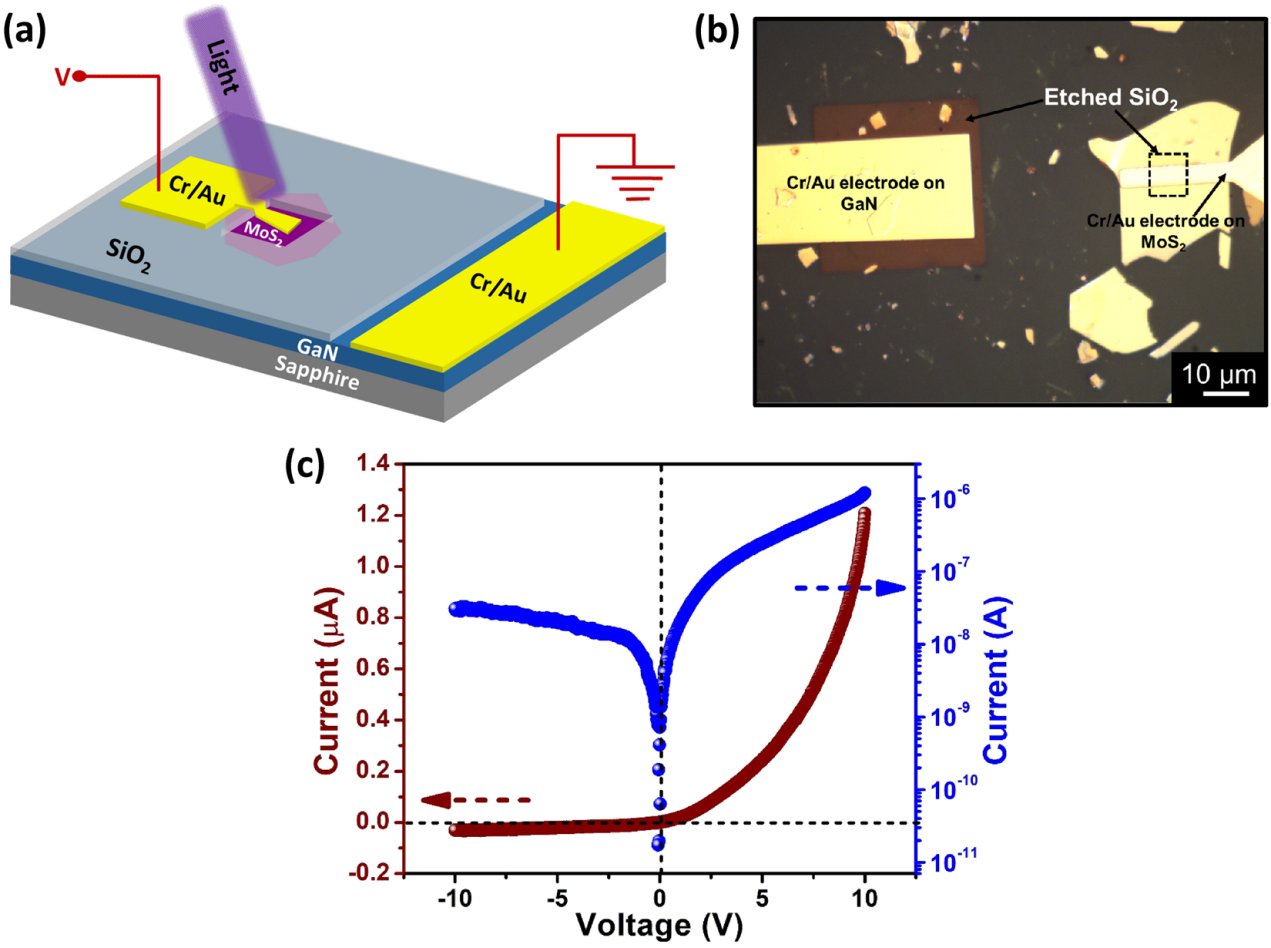
(**a**) 3D Schematic illustration of MoS_2_/GaN heterojunction device. (**b**) Optical microscope image of the fabricated heterojunction with Cr/Au (5 nm/50 nm) contacts. (**c**) Two terminal electrical measurements of the MoS_2_/GaN heterojunction diode under dark conditions showing clear current rectification.

To evaluate the electrical performance of the heterojunction, I-V measurements at room temperature were carried out. Before fabrication of the heterojunction, ohmic contacts on MoS_2_ and GaN were studied separately. Cr/Au was chosen as ohmic contact on GaN as well as on MoS_2_ (Supporting Information, Figs S2 and S3). 3D schematic diagram of the fabricated device and the optical image of the device are shown in Fig. 1(a,b). The current-voltage characteristics of the MoS_2_/GaN heterojunction under dark is shown in Fig. 1c. The I-V curve exhibits diode like rectifying behavior. Nearly ohmic behavior of Cr/Au contact to MoS_2_ as well as on GaN proves that the diode like characteristic stems from MoS_2_/GaN heterojunction.

MoS_2_ is considered to be n-type semiconductor intrinsically^39^. The n-MoS_2_/n-GaN heterojunction can be considered similar to metal-semiconductor contact and carrier transport is mainly due to majority charge carriers^40^. Since the current transport is dominated by only one type of carriers i.e. electrons similar to Schottky barrier diode, thermionic emission current equation can be applied. Therefore, barrier height and ideality factor can be calculated by slope and intercept of semi-log I-V curve:

1 $$I=A\,.{A}^{\ast }{T}^{2}\exp (\frac{-e\varphi }{kT})\times [\exp (\frac{eV}{\eta kT})-1]$$

where A is the effective area of the device ~60 μm^2^. The parameter A^*^ = 4πem*k^2^/h^3^ is the effective Richardson constant where m* is the charge carrier’s effective mass (0.57 m_o_ for MoS_2_)^41^, h and k are Planck’s constant and Boltzmann constant, respectively. ϕ is the barrier height and η is the ideality factor.

Barrier height and ideality factor can be further calculated using following equations:

2 $$\varphi =\frac{kT}{e}\,{ln}(\frac{A{A}^{\ast }{T}^{2}}{{I}_{s}})$$

where ${I}_{s}=A\,.{A}^{\ast }{T}^{2}\exp (\frac{-e\varphi }{kT})$ is the saturation current

3 $$\eta =\frac{q}{kT\{\frac{d({ln}\,I)}{dV}\}}$$

Based on the above equations, barrier height and ideality factor are estimated to be 0.50 eV and 11, respectively. Very large ideality factor (~38) is reported earlier in case of MoS_2_/GaN heterojunction as measured by CAFM^35^. The unusual large value of ideality factor is due to the interface states. Since the heterojunction diode is fabricated using scotch tape method, interface between MoS_2_ and GaN is not devoid of these interface states. Therefore, the current transport mechanism will deviate significantly from thermionic emission and other transport processes such as tunneling and recombination become dominantly, thus increasing the value of ideality factor.

### Photoresponse properties of the heterojunction

**Figure 2:**
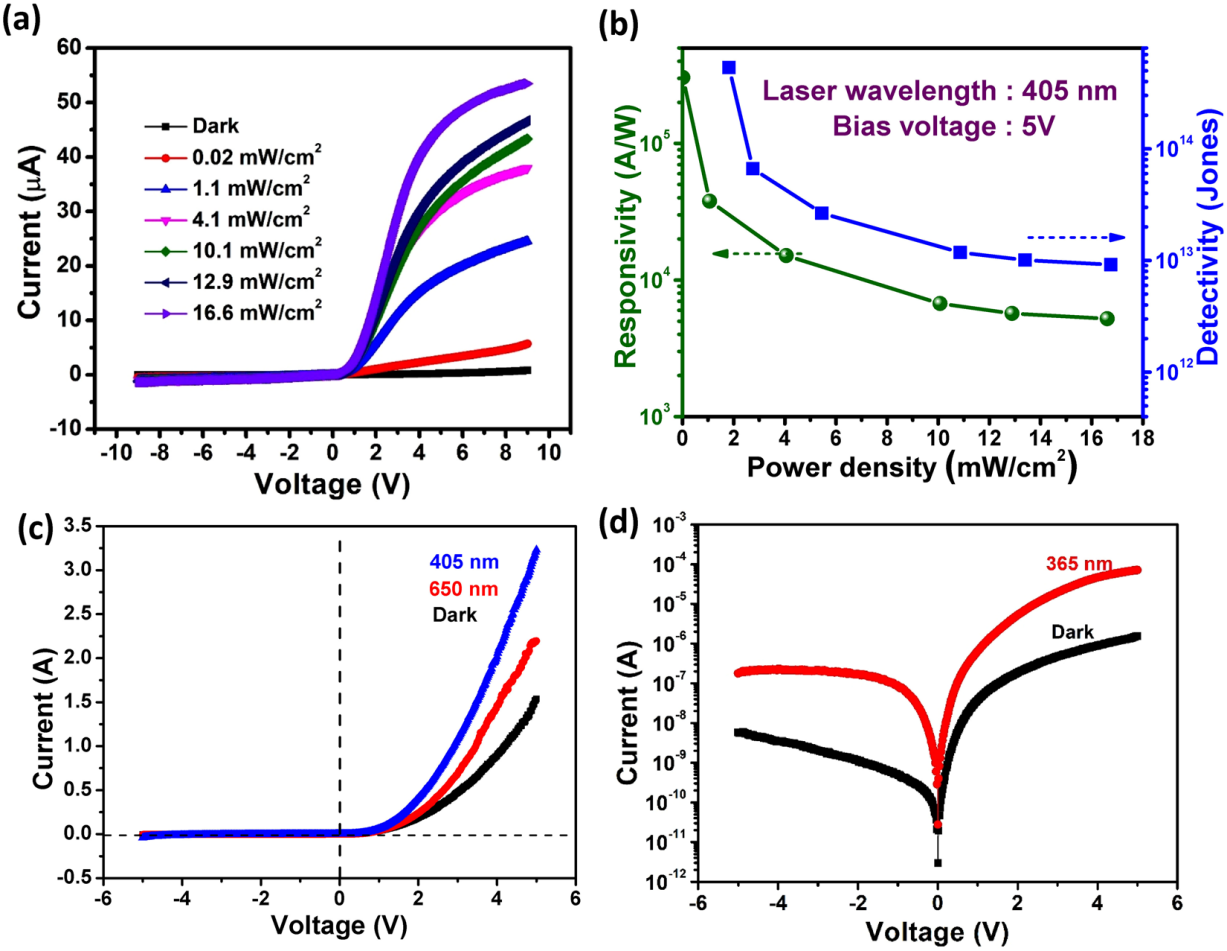
Photoresponse of MoS_2_/GaN heterojunction under illumination with 405 nm laser. (**a**) Linear plot of photoinduced behavior of MoS_2_/GaN heterojunction under different illumination intensities of 405 nm laser source. (**b**) Plots of responsivity and detectivity with light intensity. (**c**) Photoelectrical behavior of device under illumination of 650 nm and 405 nm laser. (**d**) log plot of electrical behavior of the device after exposure to UV light (365 nm).

Photoresponse properties of heterojunction are investigated by irradiating the device with 405 nm laser (Energy: 3.1 eV, greater than the MoS_2_ bandgap and lower than the bandgap of GaN). To explore the photoexcitation at the heterojunction, the photoresponse of the device was investigated with varying laser intensity ranging from 0.02 mW/cm^2^ to 16.6 mW/cm^2^ as illustrated in Fig. 2(a). Current was observed to enhance on irradiating light. At a bias of 5 V, current enhances from 2.43 × 10^−7^ A to 4.53 × 10^−5^ A at illumination intensity of 12 mW/cm^2^ giving on/off ratio of about 186.

Moreover, figure of merit parameters such as responsivity and detectivity were evaluated to check the performance of the device. Efficiency of a detector to respond to the incident light is indicated by photoresponsivity given by:

4 $${\rm{R}}=\frac{{{\rm{I}}}_{{\rm{illuminated}}}-{{\rm{I}}}_{{\rm{dark}}}}{{{\rm{P}}}_{{\rm{illuminated}}}}$$

where I_illuminated_, I_dark_ and P_illuminated_ are the current after illumination, dark current and illuminated power of laser light falling on the active area of the device, respectively^42^. Detectivity is also a critical parameter representing ability of detector to detect low optical signals and it can be estimated by^22^

5 $${\rm{D}}=\frac{{\rm{R}}\,.\,{{\rm{A}}}^{1/2}}{\sqrt{2{{\rm{eI}}}_{{\rm{d}}}}}$$

where R is the responsivity, I_d_ is the dark current which is 0.2 μA at biasing of 5 V, A is the area of the device where effective absorption of incident light occurs (~60 μm^2^) and e is the electronic charge. The corresponding change in photoresponsivity and detectivity with illuminated power is illustrated in Fig. 2b. Decrease in both responsivity and detectivity is noted with increase in power density. The decrease in responsivity and detectivity upon increasing the power density may be attributed to the trap states present at the interface of the heterojunction^31^. Maximum responsivity and detectivity are found to be 2 × 10^5^ A/W and 6 × 10^14^ jones (1 Jone = cm-Hz^1/2^/W) at power density of 0.02 mW/cm^2^ at the bias voltage of 5 V. Ultra high photoresponsivity and detectivity were observed at low intensity indicating that as fabricated device is highly sensitive to low incident optical power. Significant enhancement in detectivity is observed for the MoS_2_/GaN heterojunction; of the order of 10^14^ Jones at 0.02 mW/cm^2^ and it is higher than the value of detectivity observed in other photodetectors based on MoS_2_/3D heterojunctions. The comparative study of photodetection parameters of our fabricated device with the literature is summarized in Table 1. Also, Noise-equivalent power is given by $\sqrt{2{{\rm{eI}}}_{{\rm{d}}}}$ and for the fabricated MoS_2_/GaN heterojunction, it comes out to be 2.79 × 10^−13^ AHz^−1/2^. Moreover, the photoresponse characteristics of the heterojunction were measured with different wavelengths (650 nm and 365 nm) (Fig. 2c,d). The heterojunction also responds to 650 nm wavelength and photoelectrical characterization reveal the high sensitivity of the fabricated device in UV region (365 nm). The results give a good indication that as fabricated device is showing better switching behavior and it has potential application for photodetection.

**Table 1:** Performance comparison of our MoS_2_/GaN heterojunction based photodetector with other MoS_2_ based photodetectors.

Device	Measurement parameters	Responsivity	Detectivity	Reference
MoS_2_/GaN	λ = 405 nm, V = 5 V	10^5^ A/W	10^14^ Jones	This work
Monolayer MoS_2_ photodetector	λ = 561 nm, V = 8 V	880 A/W	—	^[1^ ^4]^
MoS_2_/Si heterojunction	λ = 808 nm, V = 0 V	300 mA/W	10^13^ Jones	^29^
MoS_2_/Si heterojunction	λ = 650 nm, V = −2V	11.9 A/W	2.1 × 10^10^ Jones	^31^
MoS_2_/hBN/GaN	Visible source, λ = 400–700 nm, V = 9 V	1.2 mA/W	—	^37^

**Figure 3:**
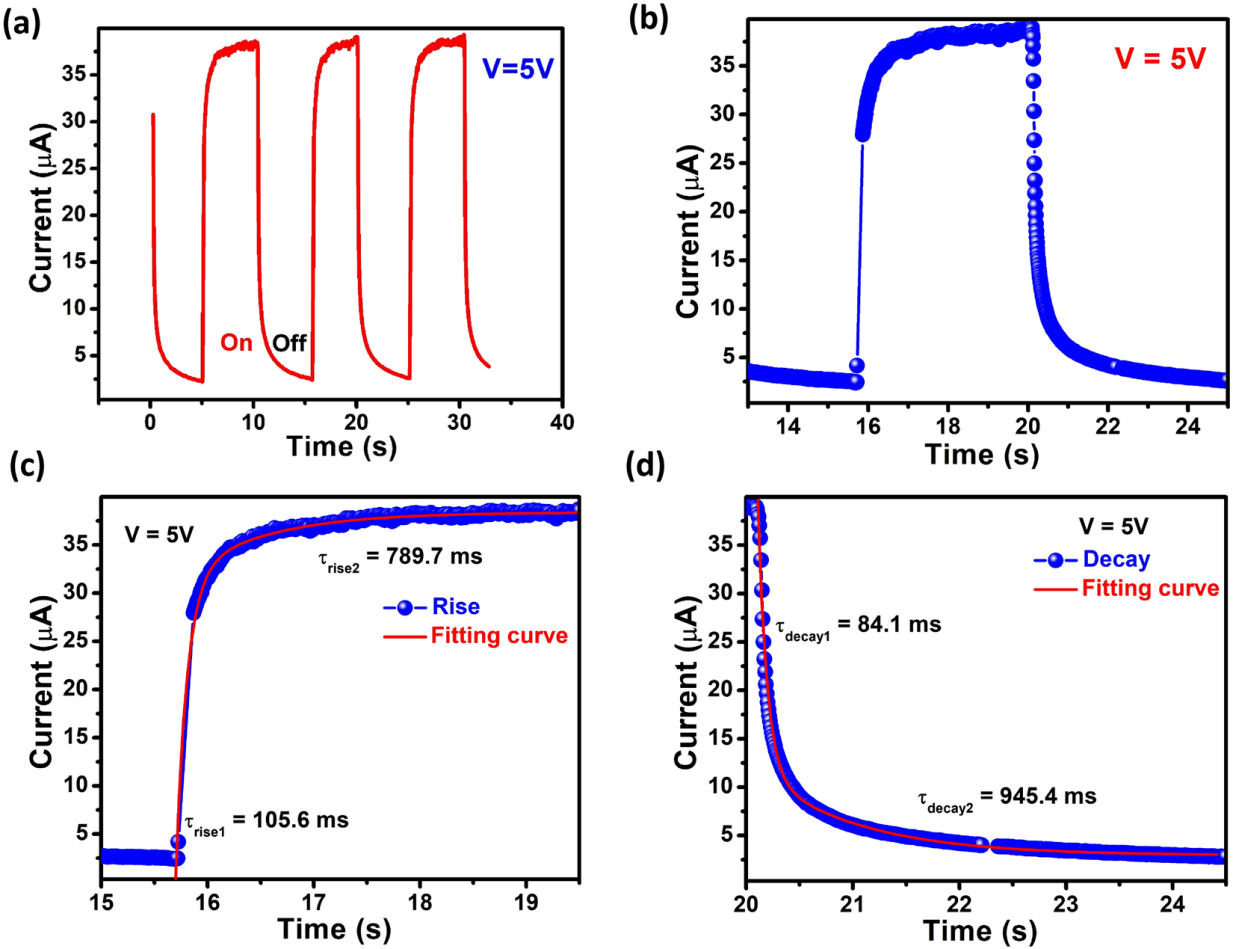
(**a**) Time dependence of photocurrent of the MoS_2_/GaN heterojunction observed by illumination via laser light (405 nm) at a bias of 5 V with fixed illumination intensity of 12 mW/cm^2^. (**b**) Magnified plot of one response cycle. (**c**) Exponential curve fitting of the rise showing fast rise time and slow rise time. (**d**) Exponential curve fitting of the decay showing fast decay time followed by slow decay (On and off represents the status when laser is on and off, respectively).

In addition, the time dependence photoresponse was investigated. Current was measured with laser on and off periodically at a constant bias of 5 V and illuminated power of 12 mW/cm^2^. Photocurrent increased when laser was turned on and it decayed on turning off the laser source (Fig. 3a). The magnified plot of the response cycle is represented in Fig. 3b. An immediate drop in current was observed followed by a slow decay. The first rise and decay time of the photocurrent were measured to be 105.6 ms and 84.1 ms, respectively (Fig. 3c,d). Fast decay is due to charge carrier relaxation due to recombination and slow decay time may be attributed to trap states present at the interface of the heterojunction.

Gain is the critical parameter in photoconduction depending on ratio of carrier lifetime to transit time. Gain of the fabricated device can be expressed as:

6 $$G=\frac{Rh\nu \,}{\eta q}$$

where R is the photoresponsivity, ν is the frequency of the incident light, q is the electronic charge and η is the external quantum efficiency^18^. Assuming ideal quantum efficiency to be 100%, Gain can be estimated to be 6.6 × 10^5^ at power density of 0.02 mW/cm^2^.

### KPFM investigation to understand the band alignment

**Figure 4:**
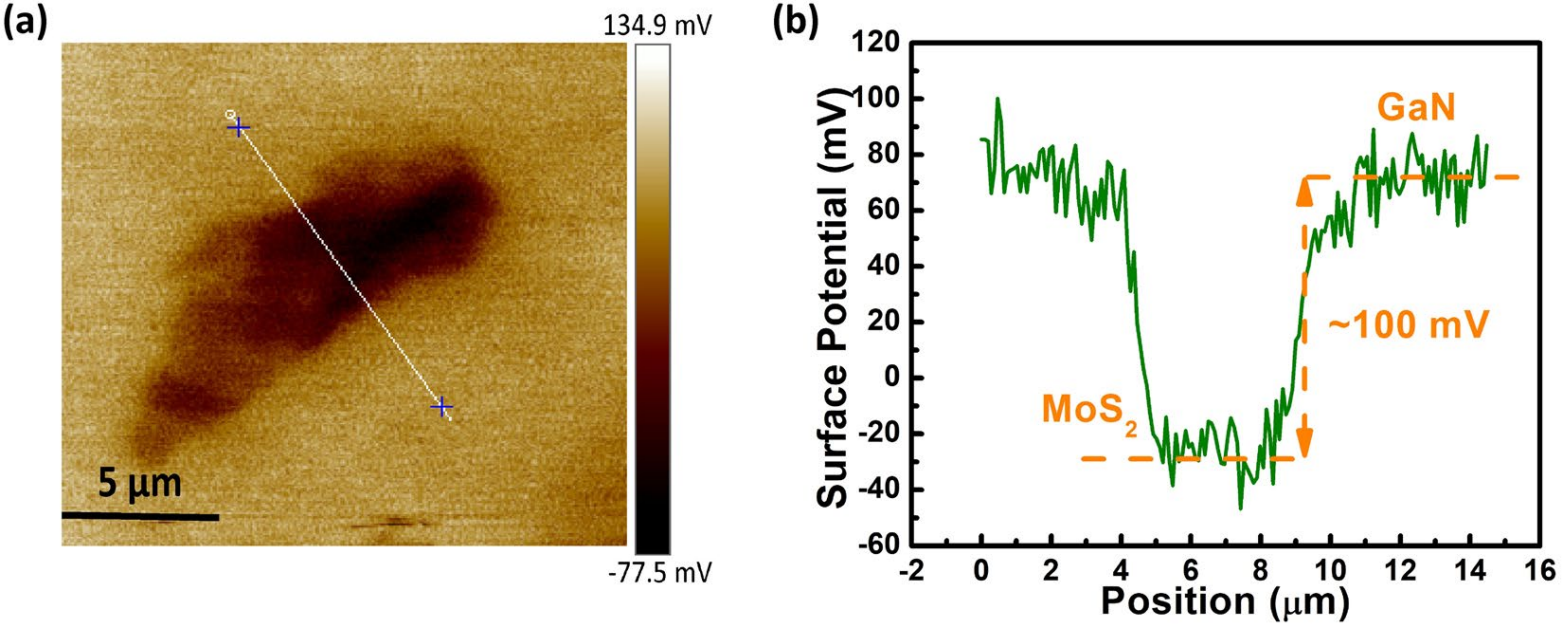
(**a**) Kelvin Probe Force Microscope (KPFM) image of MoS_2_ flake on n-GaN showing change in surface potential between MoS_2_ and GaN. (**b**) Plot of surface potential difference with lateral distance across MoS_2_/GaN interface along the line as indicated in (**a**).

In order to examine the band alignment at the MoS_2_/GaN interface, KPFM measurements were carried out as shown in Fig. 4(a,b). KPFM is a powerful technique used for surface potential mapping. KPFM has been previously employed to study the layer dependent work function of MoS_2_^43,44^ and the band alignment at 2D/3D interface^28,35^. Different methods have been employed to determine the conduction band offset of heterojunction of 2D materials with GaN^45–47^. The present investigation involves KPFM for measurement of conduction band offset. Here we experimentally investigate the change in surface potential of MoS_2_ and GaN at MoS_2_/GaN interface. Contact potential difference between the tip and the sample is given by:

$${({\rm{CPD}})}_{{{\rm{MoS}}}_{2}}=\frac{{{\rm{\varphi }}}_{{\rm{tip}}}-{{\rm{\varphi }}}_{{{\rm{MoS}}}_{2}}}{-e}\,{\rm{and}}\,{({\rm{CPD}})}_{{\rm{GaN}}}=\frac{{{\rm{\varphi }}}_{{\rm{tip}}}-{{\rm{\varphi }}}_{{\rm{GaN}}}}{-e}$$

where ϕ_tip_, ϕ_GaN_ and ${{\rm{\varphi }}}_{{{\rm{MoS}}}_{2}}$ are the work functions of the tip, GaN and MoS_2_, respectively.

The change in contact potential difference between MoS_2_ and GaN is given by:

$${\rm{\Delta }}\mathrm{CPD}={({\rm{CPD}})}_{{\rm{GaN}}}-{({\rm{CPD}})}_{{{\rm{MoS}}}_{2}}=\frac{{{\rm{\varphi }}}_{{{\rm{MoS}}}_{2}}-{{\rm{\varphi }}}_{{\rm{GaN}}}}{-e} \sim 100\,{\rm{mV}}$$

The change in surface potential of MoS_2_ and GaN is shown in Fig. 4b. The Surface potential of GaN substrate is 100 mV higher than that of the MoS_2_ flake. Hence, work function difference between GaN and MoS_2_ is extracted as

$${{\rm{\varphi }}}_{{\rm{GaN}}}-{{\rm{\varphi }}}_{{{\rm{MoS}}}_{2}} \sim 100\,{\rm{meV}}$$

Work function of GaN is observed to be greater than that of MoS_2_ with a difference of about 100 meV. In this aspect, the KPFM result gives direct evidence for junction formation between MoS_2_ and GaN substrate. Since for n-type semiconductor, relation between bottom of conduction band and fermi level is expressed as E_C_ − E_F_ = *kT* ln $(\frac{{N}_{C}}{{N}_{D}})$, therefore Conduction band offset can be further obtained by using the following equation:

7 $${{\rm{\varphi }}}_{{\rm{GaN}}}-{{\rm{\varphi }}}_{{{\rm{MoS}}}_{2}}=[{\rm{\Delta }}{E}_{c}+kT\{\mathrm{ln}\,\frac{(\frac{{N}_{C,GaN}}{{N}_{D,GaN}})}{(\frac{{N}_{C,Mo{S}_{2}}}{{N}_{D,Mo{S}_{2}}})}\}]$$

where N_D_, N_C_ and ΔE_C_ are the doping density, effective density of states and conduction band offset respectively. Doping density in n-GaN is 4.6 × 10^17^ as measured by Hall measurement technique and doping density in MoS_2_ is taken to be 10^16^ cm^−3^ ^48^

Equation (7) becomes

8 $${\rm{\Delta }}{E}_{c}=[({{\rm{\varphi }}}_{{\rm{GaN}}}-{{\rm{\varphi }}}_{{{\rm{MoS}}}_{2}})-kT\{\mathrm{ln}\,\frac{(\frac{{({{m}^{\ast }}_{GaN})}^{3/2}}{{N}_{D,GaN}})}{(\frac{{({{m}^{\ast }}_{Mo{S}_{2}})}^{3/2}}{{N}_{D,Mo{S}_{2}}})}\}]$$

Effective mass values for MoS_2_ and GaN are taken to be 0.57 m_o_ and 0.22 m_o_, respectively^41,49^. Using Equation (8), conduction band offset ΔE_c_ = 0.23 eV was estimated. According to Anderson’s rule, conduction band offset depicts the difference between electron affinities of MoS_2_ and GaN. It is evident from KPFM result that a junction barrier having value 0.23 eV exists between MoS_2_ and GaN substrate.

On the basis of these values, built in potential (V_bi_) and depletion width are calculated and therefore energy band diagram can be drawn to evolve the carrier transport.

Ideally, built in potential barrier is the difference between work functions of the semiconductors.

$${{\rm{eV}}}_{{\rm{bi}}}={{\rm{\varphi }}}_{{\rm{GaN}}}-{{\rm{\varphi }}}_{{{\rm{MoS}}}_{2}}=100\,{\rm{meV}}\,{\rm{as}}\,{\rm{measured}}\,{\rm{using}}\,{\rm{KPFM}}$$

Depletion width is given by

$${x}_{n}={\{\frac{2{\varepsilon }_{n}{\varepsilon }_{N}{N}_{dN}{V}_{bi}}{e{N}_{dn}({\varepsilon }_{n}{N}_{dn}+{\varepsilon }_{N}{N}_{dN})}\}}^{1/2}\,{\rm{and}}\,{x}_{N}={\{\frac{2{\varepsilon }_{n}{\varepsilon }_{N}{N}_{dn}{V}_{bi}}{e{N}_{dN}({\varepsilon }_{n}{N}_{dn}+{\varepsilon }_{N}{N}_{dN})}\}}^{1/2}$$

where n and N denotes MoS_2_ and GaN, respectively. *ε*_*n*_, *ε*_*N*_, N_dn_ and N_dN_ denotes the dielectric constant of MoS_2_, dielectric constant of GaN, doping density in MoS_2_ (10^16^ cm^−3^) and GaN (4.6 × 10^17^ cm^−3^), respectively. Using above values, depletion width in MoS_2_ and GaN is calculated to be 104 nm and 2.26 nm, respectively.

Furthermore, built in potential barrier in both the regions can be calculated using following equation:

9 $$\begin{array}{c}{V}_{bi}={V}_{bin}+{V}_{biN}=\frac{e{N}_{dn}{{x}_{n}}^{2}}{2{\varepsilon }_{n}}+\frac{e{N}_{dN}{{x}_{N}}^{2}}{2{\varepsilon }_{N}}\\ {V}_{bin}\,=97\,{\rm{m}}{\rm{V}}\,{\rm{a}}{\rm{n}}{\rm{d}}\,{V}_{bin}=2.23\,{\rm{m}}{\rm{V}}\end{array}$$

Built in potential barrier in MoS_2_ and GaN region are calculated to be 97 mV and 2.23 mV, respectively. The calculated values of depletion width and built-in potential barrier in both the regions help in evolving the band diagram and the charge transport.

## Discussion

**Figure 5:**
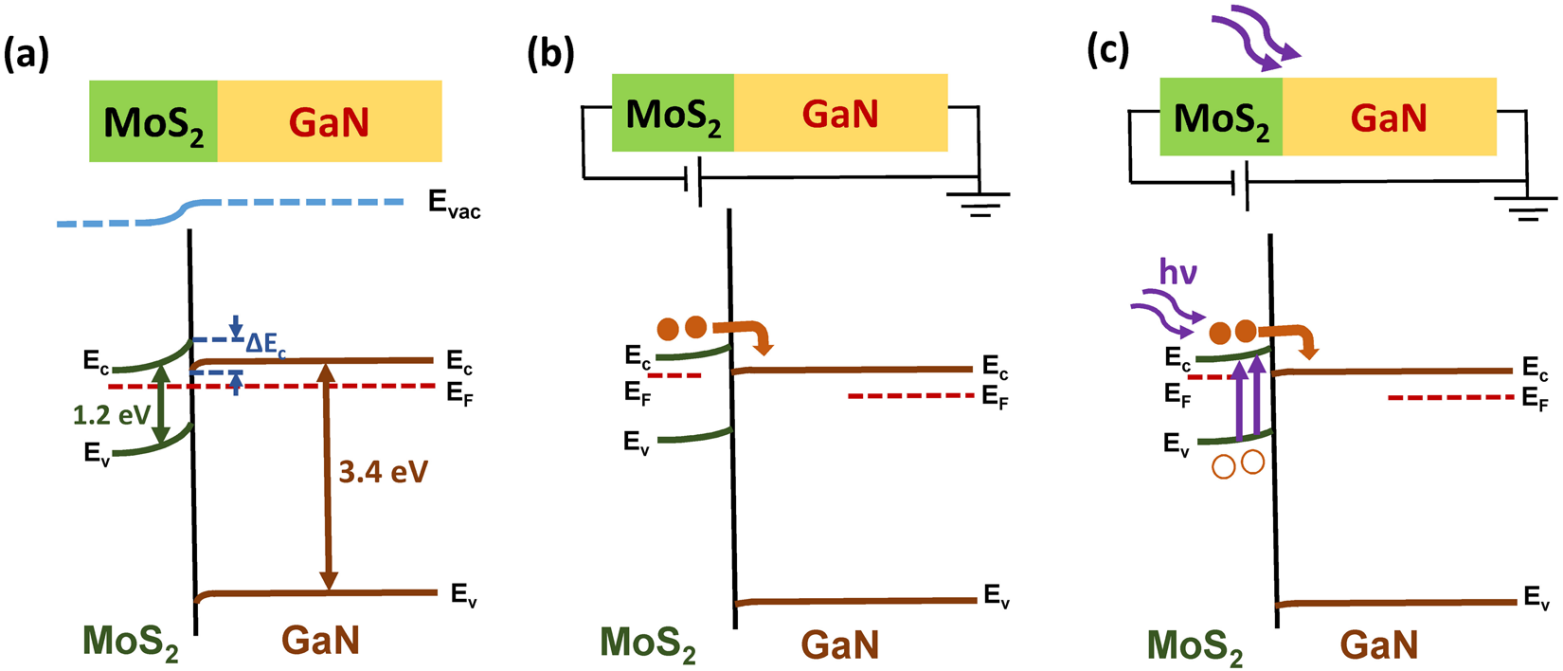
Energy band diagram of the heterojunction. (**a**) After contact (zero bias). (**b**) Forward bias (negative bias is given to MoS_2_ with respect to GaN). (**c**) Forward bias under photoexcitation with laser wavelength 405 nm. E_c_, E_v_, E_F_ and E_vac_ represent bottom of conduction band, top of valence band, Fermi level and vacuum level, respectively. Band gap of MoS_2_ and GaN is 1.2 eV and 3.4 eV, respectively. ΔE_c_ represents the conduction band offset.

In order to understand the carrier transport and photoresponse of the device, a possible mechanism is proposed based on type II band alignment as illustrated in Fig. 5(a–c). Band diagram is drawn based on the above calculated parameters based on KPFM such as built in potential, depletion width and conduction band offset. Figure 5a represents the schematic of the band alignment under zero bias condition demonstrating type- II heterojunction. Under equilibrium condition, when MoS_2_ is in contact with GaN, band bending occurs in order to align the Fermi level. Since Fermi level of MoS_2_ is at higher energy level than that of GaN, electrons in MoS_2_ side will tend to move to GaN forming a built in potential at the interface. MoS_2_ acquires a depletion region whereas GaN acquires an accumulation region near the interface. Depletion width and built in potential in MoS_2_ region is larger than that of GaN region as calculated above. When MoS_2_ is given negative voltage with respect to GaN, higher current is observed compared to reverse bias case. Schematic of energy band structure on biasing is illustrated in Fig. 5b. When MoS_2_ is forward biased with respect to GaN, band edge of MoS_2_ raises and that of GaN lowers down. Electrons from MoS_2_ region can transport to GaN region due to decrease of effective barrier for flow of electrons from left side to right side giving high value of current. However, when MoS_2_ is reverse biased with respect to GaN, there is less probability for electrons in MoS_2_ region to move to GaN region because of relatively higher barrier. Hole current is not considered due to essential lack of holes.

Under illumination with light energy (405 nm) higher than bandgap of MoS_2_, electron hole pairs are generated preferentially in MoS_2_ due to lower incident excitation energy than bandgap of GaN as shown in Fig. 5c. When negative voltage is applied to MoS_2_, photocurrent is enhanced due to transport of photogenerated electrons from MoS_2_ region to GaN region. The observed lesser current in reverse biased case is due to higher barrier formation for photogenerated carriers to flow from MoS_2_ to GaN. However, when the device is illuminated with 365 nm laser, large photocurrent is observed. Electrodes on MoS_2_ and GaN are separated by a distance of nearly 60 μm whereas spot size of the incident laser is 2 mm. The incident light is illuminating both GaN and MoS_2_. Since 365 nm wavelength is corresponding to bandgap of GaN, electron-hole pairs are generated in GaN also leading to significant enhancement in current. Both MoS_2_ and GaN are contributing to the total current leading to large enhancement in the photocurrent.

## Conclusions

MoS_2_/GaN diode which is a type-II heterojunction has been fabricated. The type II band alignment of 2D MoS_2_/GaN heterojunction has been investigated by measuring change in surface potential and corresponding change in work functions of MoS_2_ and GaN. The conduction band offset of 0.23 eV was extracted using KPFM. From the current-voltage measurements, the heterojunction exhibited diode like behavior with barrier height of 0.50 eV in dark. In addition, the photoresponse behavior of the device was explored and the device was highly sensitive to 405 nm laser. The as fabricated heterojunction exhibited excellent optoelectronic performance such as ultrahigh photoresponsivity as 10^5^ A/W, gain as 10^5^ and detectivity of the order of 10^14^ Jones. These results show that 2D MoS_2_/GaN can be used in efficient photodetection applications. Our results can pave the way in designing the optoelectronic devices based on integration of low dimensional materials with conventional 3D semiconductors.

## Experimental Section

The MOVPE grown Gallium Nitride (GaN) epitaxial film on c-plane sapphire substrate with 3 μm thickness has been used for MoS_2_/GaN heterojunction. The GaN layer exhibits n-type behavior, sheet resistance of about 285 Ω/square, carrier concentration of 4.6 × 10^17^ cm^−3^ and Hall electron mobility of about 160 cm^2^/Vs at room temperature, as measured by Ecopia Hall measurement set up (HMS 5000). The samples were ultrasonically cleaned in acetone followed by iso-propanol and De-ionized water (DI-Water) for 5 minutes each, to remove the organic contaminants. In order to etch the native oxide layer from the surface, samples were dipped in the solution of HCl: H_2_O in the ratio of 1:2 for 30 s. Samples were again rinsed with DI water and thereafter dried with nitrogen gun.

**Figure 6:**
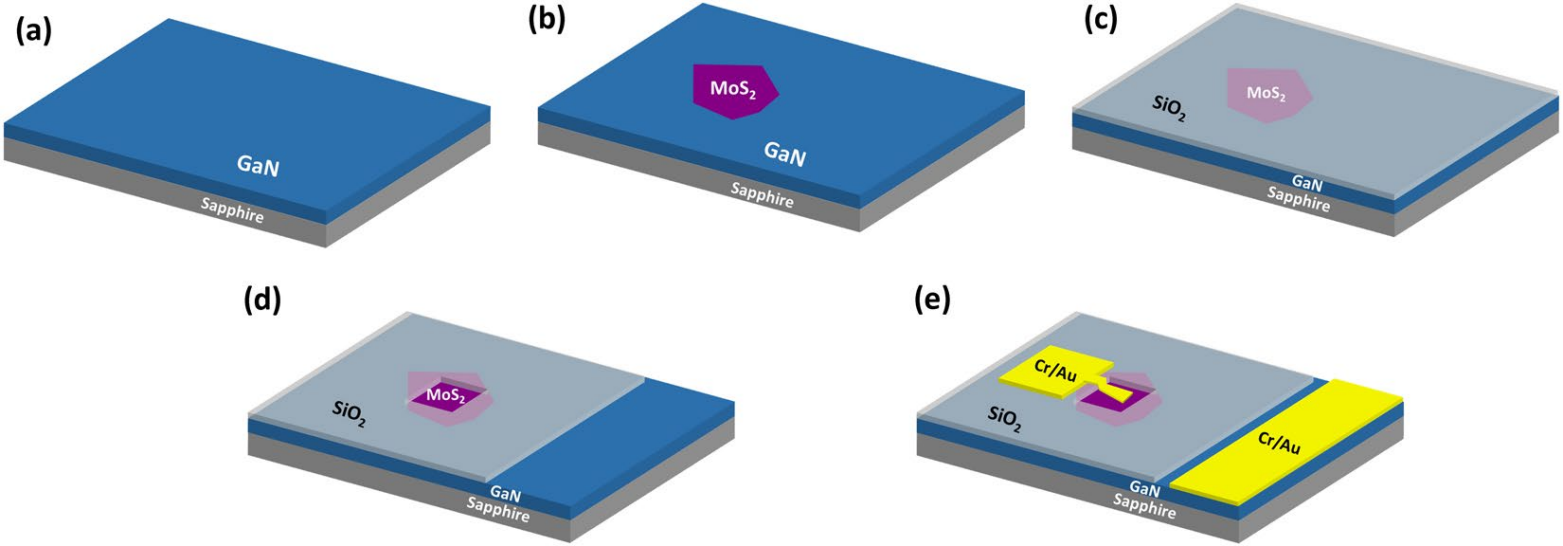
Process flow of the fabrication process. (**a**) GaN/Sapphire substrate. (**b**) Transfer of MoS_2_ flakes on GaN/Sapphire substrate. (**c**) Deposition of SiO_2_ on the template for insulation of electrodes. (**d**) Selective etching of SiO_2_ over MoS_2_ and GaN. (**e**) Electrode deposition on MoS_2_ and GaN.

MoS_2_ crystal was purchased from SPI supplies. For the fabrication of MoS_2_-GaN heterojunction, 2D MoS_2_ flakes were transferred on GaN/sapphire from MoS_2_ crystal using mechanical exfoliation method (Fig. 6a,b). Raman measurement was conducted to confirm the MoS_2_ flake using micro- Raman system (Horiba LabRAM HR evolution) with laser excitation wavelength of 514 nm. KPFM measurements were performed using Bruker multi mode Atomic Force Microscope in tapping mode. Pt coated Si tip was used to scan the samples and samples were grounded during the measurements.

After transfer of MoS_2_ on GaN, SiO_2_ of thickness 100 nm was deposited on MoS_2_/GaN sample using sputtering as an insulating layer (Fig. 6c). PMMA resist was spin coated on the template. Windows were opened on top of MoS_2_ (8 μm × 8 μm) and GaN (40 μm × 40 μm) using Electron beam lithography (Model No: eLine plus from Raith GmbH). SiO_2_ was etched away within the opened window by dipping the sample in 10% HF for 10 seconds (Fig. 6d). After removing the remaining resist in acetone, again electron beam lithography was used to pattern the electrodes over MoS_2_ and GaN. Finally, metal contacts were deposited using sputtering system followed by lift off of resist in acetone. Cr/Au (5 nm/50 nm) was chosen as top electrode on MoS_2_ as well as bottom electrode on GaN (Fig. 6e). Current-voltage characteristics of the device were measured using DC probe station (EverBeing-EB6) and Semiconductor Characterization System (Keithley: SCS-4200) under dark and illuminated conditions. Tungsten tips were used to probe the electrodes. For illumination of the device, laser source was used. Photoresponse of the device was measured at different power densities of the light.

## Electronic supplementary material

Supplementary Information
