# Supplementary material for: Understanding of MoS2/GaN Heterojunction Diode and its Photodetection Properties
Source: Sci Rep. 2018 Aug 7;8:11799. doi: 10.1038/s41598-018-30237-8 (PMC6081413; doi:10.1038/s41598-018-30237-8)
Supplement: Supplementary file 1 — Supplementary Information [file 41598_2018_30237_MOESM1_ESM.pdf]

Supporting Information

Understanding of MoS_2/GaN Heterojunction Diode and its Photodetection Properties

Monika Moun*, Mukesh Kumar, Manjari Garg, Ravi Pathak and Rajendra Singh
Department of Physics, Indian Institute of Technology Delhi, New Delhi, 110016, India
*monikamoun18@gmail.com

Table of contents:

S1. Raman spectra of multilayer MoS_2

S2. Electrical characterization of ohmic contact to n-GaN

S3. Fabrication and electrical characterization of Cr/Au contact to MoS_2

S4. Photocurrent vs power density of the heterojunction

S1

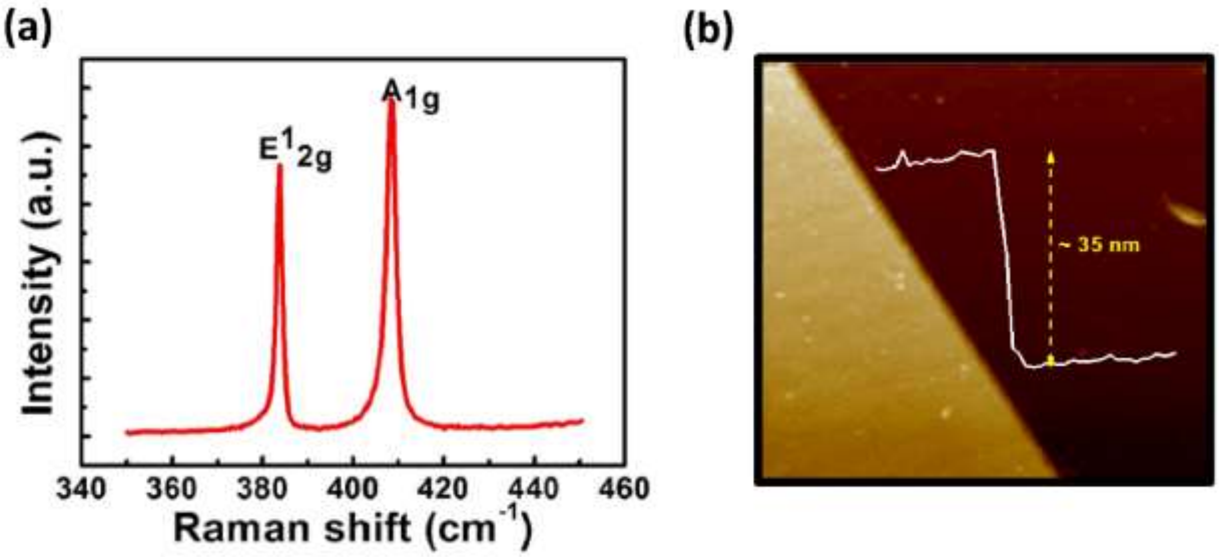

Figure S1. (a) Raman spectra of exfoliated MoS_2 flake taken using 532 nm laser indicating in plane E^1_{2g} and out of plane A_{1g} raman mode **(b)** AFM image of the exfoliated flake.

S2

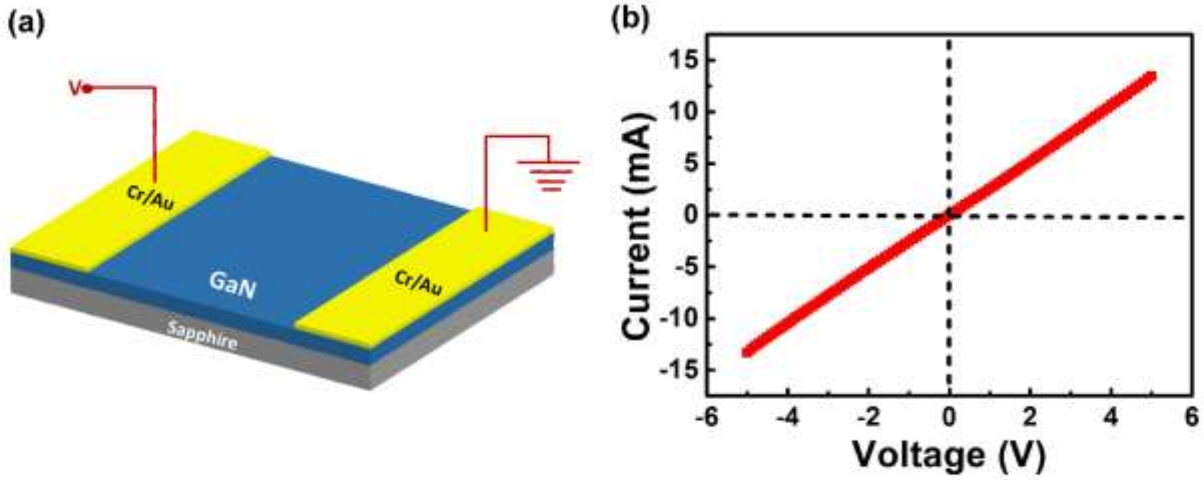

Figure S2. Fabrication of ohmic contact to n-GaN a) Schematic diagram of metal contacts to n-GaN b) Current–voltage characteristics of ohmic contacts to n-GaN. I-V curve shows the ohmic behavior of n-GaN with Cr/Au (5nm/50nm) contacts.

S3

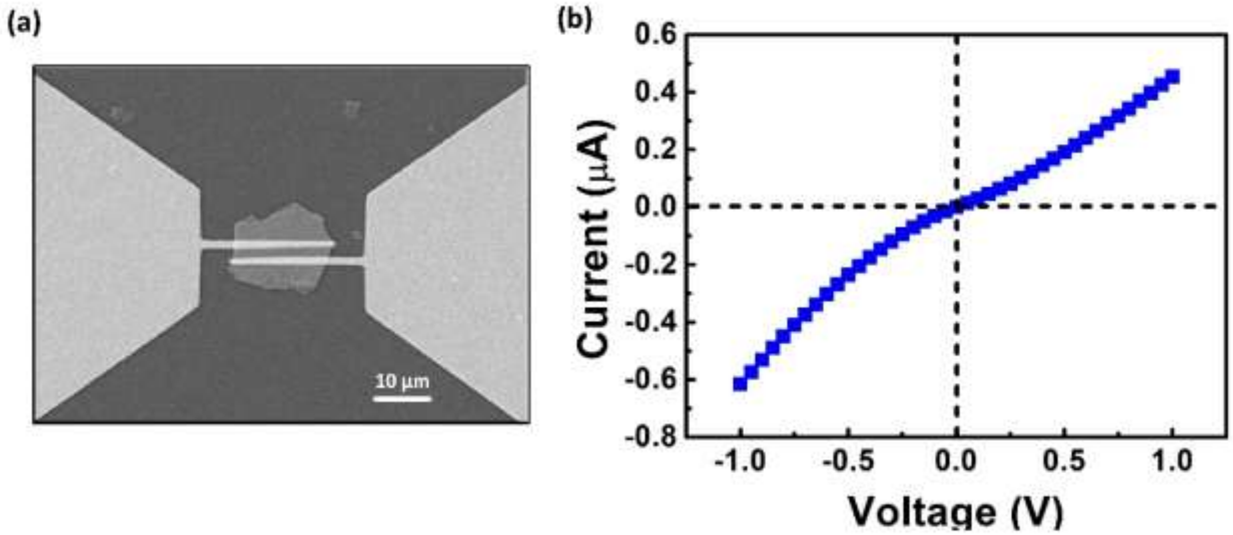

Figure S3. Fabrication of metal contacts to MoS_2 flake a) SEM image of fabricated Cr/Au (5nm/50nm) contacts on MoS_2 flake fabricated using e-beam lithography b) Electrical characterization of Au/Cr/MoS_2 showing nearly ohmic behavior.

Before fabrication of the heterojunction, ohmic contact on MoS_2 and GaN was studied separately. Cr/Au was chosen as ohmic contact on GaN[1] as well as on MoS_2[2]. Figures S2 and S3 shows the ohmic contacts to GaN and MoS_2, respectively. Cr/Au shows nearly ohmic behavior with MoS_2. The diode behavior of the MoS_2/GaN heterojunction is due to type II band alignment of MoS_2/GaN heterojunction.

S4

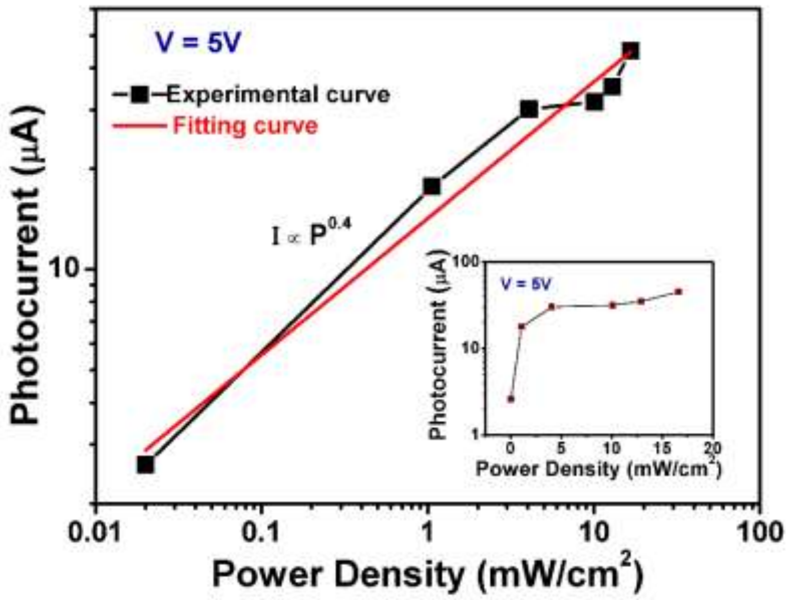

Figure S4. Photocurrent of the MoS_2/GaN heterojunction under illumination with 405 nm laser as a function of power density. The inset figure shows the linear plot.

Enhancement in photocurrent is observed with increasing laser power density as shown in Figure S4. Photocurrent of the device can be related to the incident power by the relation $I_{ph} = AP_{in}^{\alpha}$

Where I_{ph} is the photocurrent, P_{in} is the incident power and α is the exponential factor determining the behavior of photocurrent with incident light[3]. In the present case α comes out to be 0.4. Similar

results have been reported previously for MoS_2/Si heterojunction[4,5]. Factors like the trap states present at the interface and recombination process may cause the deviation of the law from unity[5].

References:

1 Lee, M. L., Sheu, J. K. & Hu, C. C. Nonalloyed Cr/Au-based ohmic contacts to n-GaN. *Appl Phys Lett* **91,** 182106 (2007).

2 Choi, M. S. *et al.* Lateral MoS_2 p-n Junction Formed by Chemical Doping for Use in High-Performance Optoelectronics. *Acs Nano* **8**, 9332-9340 (2014).

3 Yang, S. X. *et al.* Layer-dependent electrical and optoelectronic responses of $ReSe_2$ nanosheet transistors. *Nanoscale* **6**, 7226-7231 (2014).

4 Wang, L. *et al.* MoS_2/Si Heterojunction with Vertically Standing Layered Structure for Ultrafast, High-Detectivity, Self-Driven Visible-Near Infrared Photodetectors. *Adv Funct Mater* **25**, 2910-2919 (2015).

5 Zhang, Y. *et al.* In Situ Fabrication of Vertical Multilayered MoS_2/Si Homotype Heterojunction for High-Speed Visible-Near-Infrared Photodetectors. *Small* **12**, 1062-1071 (2016).
